# Wallerian degeneration of the middle cerebellar peduncles secondary to pontine infarction, case report, and review of literature

**DOI:** 10.1016/j.radcr.2025.04.003

**Published:** 2025-05-06

**Authors:** Zaina Brinji, Dania G Felemban, Basem Bahakeem, Mohammed H Almatrafi

**Affiliations:** a Department of Radiology, King Abdullah Medical City, Makkah, Saudi Arabia; b Department of Medicine, Umm Al-Qura University, Makkah, Saudi Arabia; c Medicine and Surgery, Umm Al-Qura University, Makkah, Saudi Arabia

**Keywords:** Wallerian degeneration, Pontine infarction, Ischemic stroke, Atherosclerosis, Cerebellar symptoms

## Abstract

Wallerian degeneration (WD) is a process of axonal degradation that occurs downstream from a primary injury site and is often seen after strokes affecting the brainstem. This case report documents WD of the bilateral middle cerebellar peduncles (MCPs) in an 81-year-old woman following chronic left paramedian pontine infarction (PPI). An ischemic stroke affecting the pons can trigger downstream axonal degeneration, known as Wallerian degeneration, which involves the structural breakdown of axons distal to the injury site. In this patient, magnetic resonance imaging (MRI) revealed symmetrically increased T2 signal intensity in the MCPs, indicative of WD associated with a known PPI. Although uncommon, it is essential to recognize WD in this location to distinguish it from new ischemic lesions. While the WD of MCPs has not been strongly linked to specific deficits, this phenomenon advances our understanding of the secondary effects of pontine strokes (PS). Detailed documentation of such cases helps improve diagnostic accuracy and prevents misinterpretation of expected postinfarct changes in neuroimaging. This report provides a valuable example of increasing awareness of WD involving MCPs due to PPI.

## Introduction

Damage to medial cerebellar peduncles (MCPs), although relatively rare, is a critical pathological condition associated with a range of medical issues, including ischemic strokes [1,2]. The narrowing of large arteries often triggers these strokes owing to atherosclerotic plaque buildup [1,3]. One of the critical processes involved in the aftermath of such neural injury is Wallerian degeneration (WD), which describes the degeneration of axons distal to the injury site [2,4]. This leads to scarring and atrophy within neural pathways and is an early complication typically beginning within the first week poststroke, prominently affecting the corticospinal tracts [4,5].

WD is particularly prevalent in the intricate network of nerve fibers within the pons, especially following an infarct at its base [4,5]. This can result in symmetric and bilateral damage to the MCPs [2,4]. WD manifests on magnetic resonance imaging (MRI) as T2 hyperintensity and is clinically correlated with cerebellar symptoms, including ataxia, dysmetria, and dysarthria. While unilateral pontine lesions may cause WD in the ipsilateral MCP, bilateral pontine lesions often lead to damage on both sides [4,5].

The etiology of MCP lesions is diverse, not only due to stroke but also due to neurodegenerative diseases such as multiple system atrophy, inflammatory conditions such as neuromyelitis optica, exposure to toxins such as heroin, and primary central nervous system tumors such as lymphomas [6,7]. The similarity of bilateral MCP lesions across different conditions, including demyelinating processes, metabolic disorders, and toxic injuries, poses a significant challenge for accurate diagnosis [6,8].

Therefore, clinicians must maintain a high level of vigilance and have an in-depth understanding of these conditions for effective management. The literature contains only a handful of case reports on WD in MCPs secondary to paramedian pontine infarction (PPI), underscoring its rarity and the importance of its recognition in clinical practice. Our case report describes an elderly female patient who developed WD in bilateral MCPs following chronic left PPI, illustrating the characteristic features of this condition. By reviewing such cases and the relevant literature, we aimed to shed light on the pathophysiology, diagnostic imaging findings, and clinical implications of WD secondary to PS. Enhancing awareness and knowledge of this uncommon but significant condition is imperative to improve patient care for those affected by PPI.

## Case report

An 81-year-old Saudi female presented to the emergency department with acute-onset chest pain radiating to her left shoulder. The pain started approximately 2 hours before the presentation and was described as pressure-like, rated 7/10 in severity, with no alleviating or aggravating factors.

She presented with a significant past medical history of type 2 diabetes mellitus managed with metformin 1000 mg twice daily, essential hypertension controlled with amlodipine 5 mg daily, and hypothyroidism maintained with levothyroxine 100mcg daily. She had experienced right facial palsy 2 years before the presentation, although the workup remained incomplete. Her family history was significant for cardiovascular disease, with her father deceased from myocardial infarction at age 75 years and her mother from stroke at age 82 years. She was a retired schoolteacher and a nonsmoker, living with her daughter and independent in activities of daily living.

Upon examination, her vital signs showed mild hypertension with a blood pressure of 158/92 mmHg, heart rate of 88 beats/min, respiratory rate of 18 breaths/min, temperature of 36.8°C, and oxygen saturation of 98% on room air. She was alert and oriented, with regular cardiac rhythm and clear bilateral breath sounds. Neurological examination revealed residual right facial weakness (House-Brackmann grade III) but normal power in all limbs, intact sensation, deep tendon reflexes, and no cerebellar or meningeal signs.

Laboratory investigations revealed a normal complete blood count with hemoglobin 13.2 g/dL, white blood cells 8.4 × 10^9/L, and platelets 245 × 10^9/L. Cardiac marker levels were elevated, with troponin I at 0.9 ng/mL and CK-MB levels of 28 U/L. The basic metabolic panel and coagulation profile were within the normal limits. Her HbA1c level was mildly elevated at 7.2%, while thyroid function tests were normal with a TSH of 2.8 mIU/L and free T4 of 1.2 ng/dL.

**Fig. 1:**
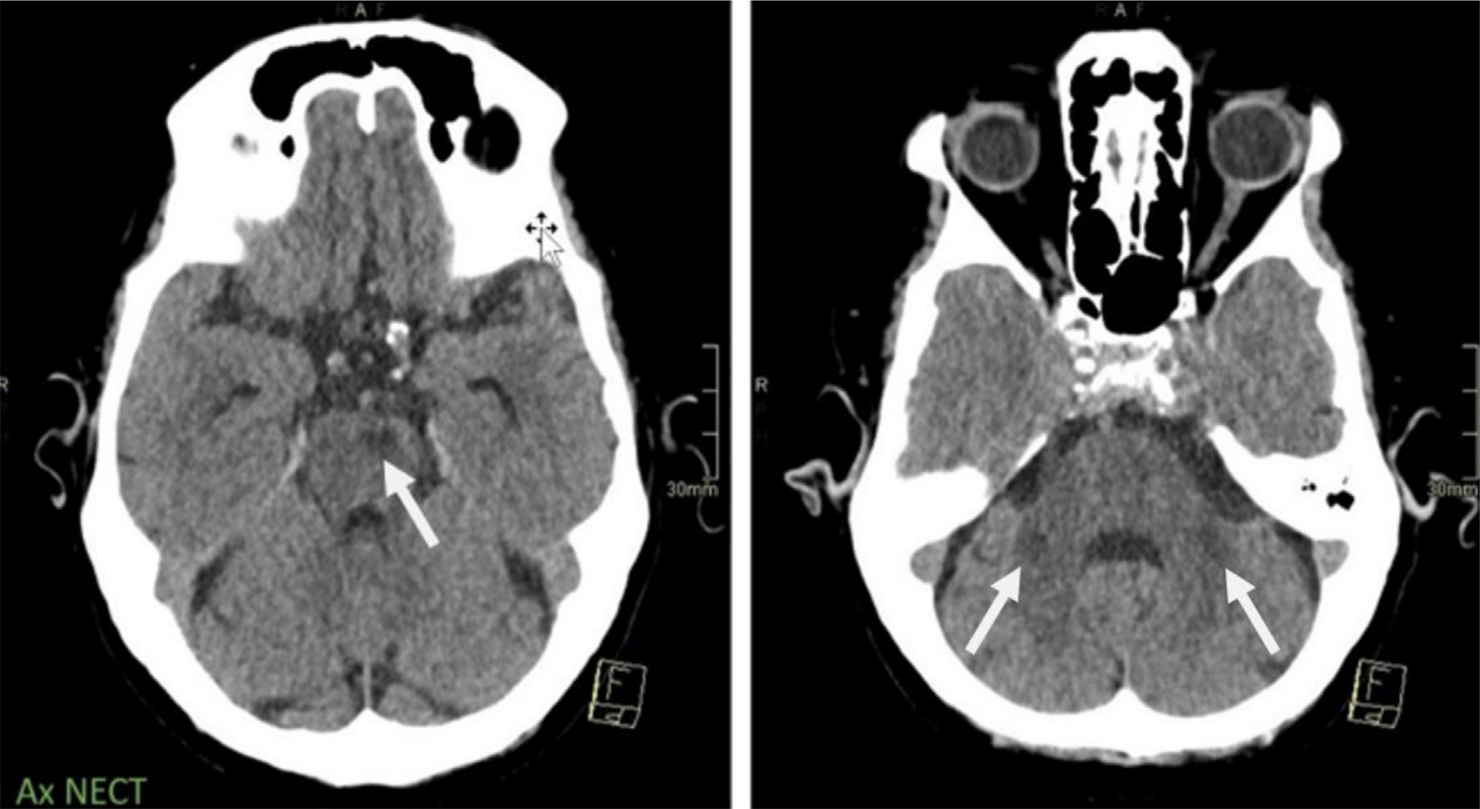
Axial noncontrast CT scan of the brain showing a small hypodensity in the left paramedian pontine region (white arrow) representing a chronic infarct (left image). Note the bilateral symmetrical hypodensities in the middle cerebellar peduncles (black arrows), consistent with Wallerian degeneration (right image).

The patient also had a history of right facial palsy 2 years prior, for which no clear etiology had been determined, and no other focal neurological deficits were noted at the time of admission. Computed tomography (CT) of the brain showed a small hypodensity in the left paramedian pontine region, indicative of an infarct of indeterminate age. Additionally, bilateral nonspecific symmetrical hypodensities of MCPs were observed (Fig. 1).

**Fig. 2:**
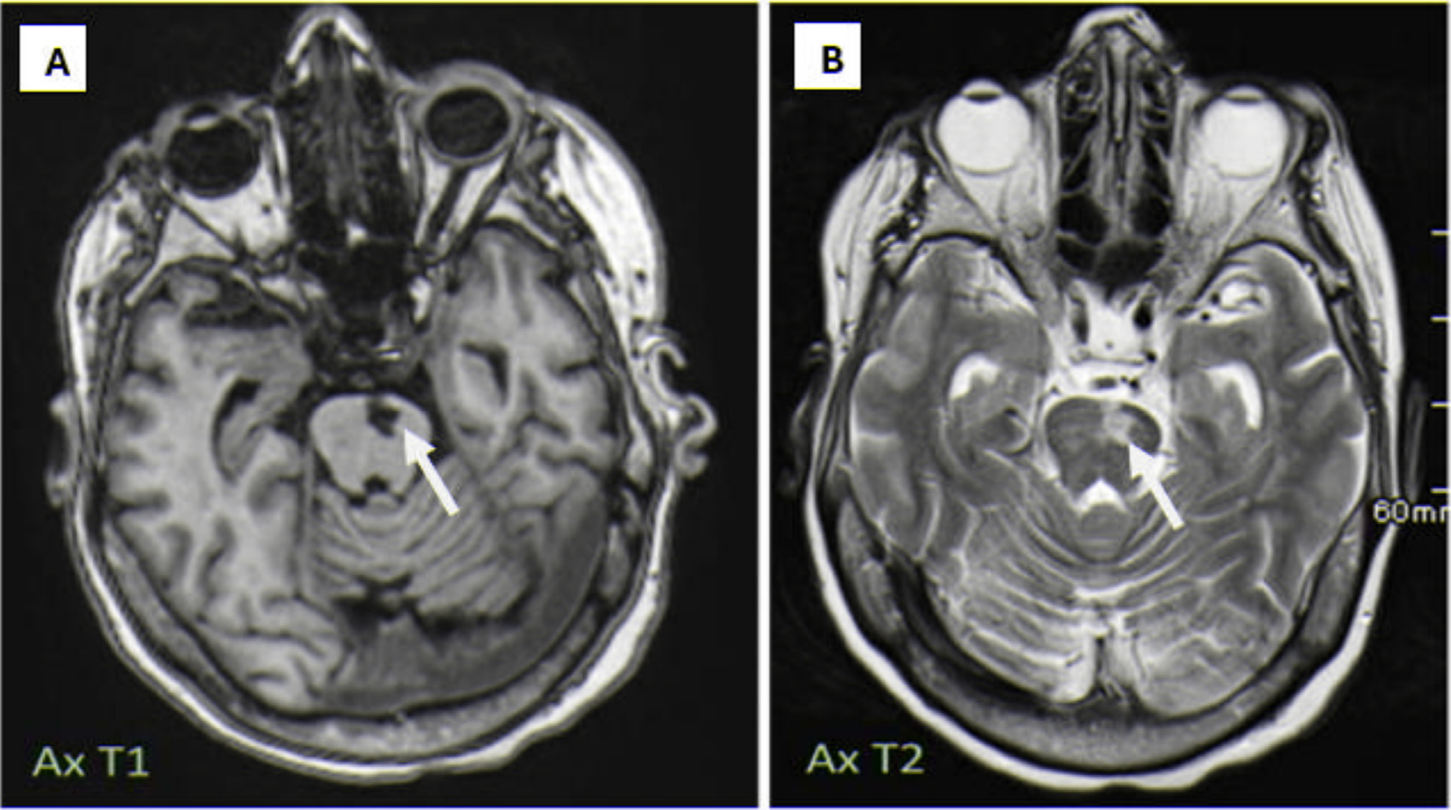
Brain MRI demonstrating chronic left paramedian pontine infarction. (A) Axial T1-weighted image showing a hypointense signal in the left paramedian pons (white arrow). (B) Axial T2-weighted image revealing a hyperintense signal in the corresponding region (white arrow), confirming the chronic nature of the infarct.

Subsequent brain MRI revealed chronic infarction in the left paramedian pontine region (Fig. 2).

**Fig. 3:**
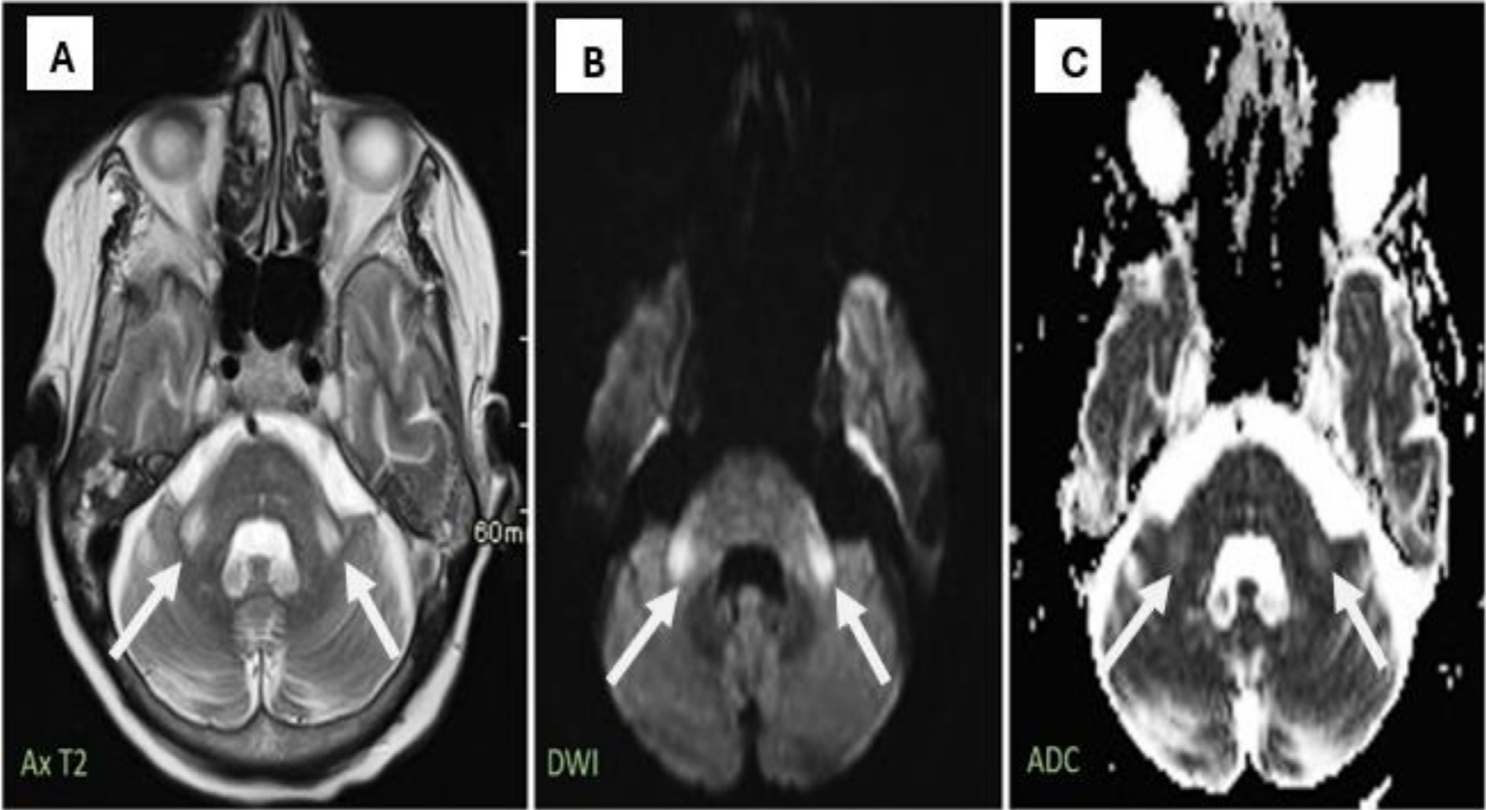
MRI findings of bilateral middle cerebellar peduncle Wallerian degeneration. (A) Axial T2-weight image (Ax T2) showing a symmetrical hyperintense signal in the bilateral middle cerebellar peduncles (white arrows). (B) DWI sequence and (C) ADC map revealing corresponding mildly increased signal intensity, typical of chronic Wallerian degeneration rather than acute infarction.

Furthermore, symmetrical T2 hyperintensity in the bilateral MCPs was noted, along with a minor increase in diffusion-weighted imaging (DWI) and apparent diffusion coefficient (ADC) signal intensity (Fig. 3). These findings suggest WD.

## Management and outcome

Based on the presenting symptoms and elevated cardiac markers, the patient was diagnosed with NSTEMI and admitted to the Cardiac Care Unit. Initial management included heparin infusion, aspirin 300 mg loading dose, and continuation of regular medications. Given her history of facial palsy and incidental imaging findings, further neurological evaluation was performed. A brain CT scan revealed a small hypodensity in the left paramedian pontine region and bilateral nonspecific symmetrical hypodensities of the MCPs. Follow-up brain MRI revealed chronic infarction in the left paramedian pontine region, symmetrical T2 hyperintensity in the bilateral MCPs, and a minor increase in DWI and ADC signal intensity. During hospitalization, her chest pain resolved with medical management, and cardiac catheterization revealed nonobstructive coronary artery disease. The patient remained neurologically stable and was discharged after 5 days of dual antiplatelet therapy (aspirin 81 mg and clopidogrel 75 mg daily), atorvastatin 40 mg daily, and adjusted doses of previous medications. At the 2-week follow-up, she reported no recurrence of chest pain or new neurological symptoms and was scheduled for regular cardiology and neurology follow-up.

## Literature review

WD is a pathological degenerative process that follows neuronal injury, especially in conditions such as stroke and infarction. Thus, understanding WD following pontine infarction is paramount for better diagnosis and management of the patient. However, WD of the MCP is a secondary consequence of pontine infarction because the pontocerebellar tracts are disrupted. Gala et al. (2013) documented that MRI diagnosed acute PI and WD, proving that transient restricted diffusion in the MCPs might mimic new infarcts [9]. Apostolakopoulou et al. (2022) described bilateral symmetric T2 hyperintense MCP lesions in chronic paramedian pontine infarction, stressing that this finding should raise some suspicion of WD. Where unfavorable outcomes were reported [10], Yin et al. (2019) described asymptomatic bilateral MCP degeneration as meaning that there had indeed been functional compensation [11].

Characteristic imaging patterns have been reported in several studies. In multiple patients, Shen et al. demonstrated that bilateral symmetric MCP hyperintensities on T2/FLAIR sequences can indicate pontine infarction as a reliable marker for WD [12,13]. These markers can be considered reliable for WD. Moreover, Uchino et al. (2004) describe distinctive WD patterns as symmetrical hyperintense MCP lesions following ventromedial pontine infarction and ipsilateral lesions after ventrolateral pontine infarction [14]. Hyperintense MCP lesions on T2-weighted imaging in a case of ventromedial pontine infarction and contralateral lesions in a ventrolateral infarction. An alternative degeneration pattern on a single side came with their case report in 2023, and the anatomy of the pontocerebellar tract provided an anatomical explanation [15]. Such degeneration concurrently occurs with hypertrophic olivary degeneration and bilateral MCP degeneration in an ipsilateral pontine infarction, as reported by Bao et al. [16].

The pathophysiological mechanisms of WD remain controversial. While some researchers have postulated degeneration through the dentatorubral and dentatothalamic pathways, which are trans-synaptically mediated [17,18], others believe that it is vasogenic edema followed by compression in large pontine infarcts [10,18]. However, these theories are not favored for WD of small pontine lesions [19].

These other conditions include but are not limited to, neurodegenerative fragile X-associated tremor/ataxia syndrome, multiple system atrophy, vascular posterior reversible encephalopathy syndrome, and bilateral anterior inferior cerebellar artery infarction. Infections have also been reported in HIV encephalopathy and JC viruses. The recognition of the disease is important, mainly to rule out new ischemic lesions or other pathologies [20,21].

Indeed, WD in MCP following pontine infarcts is not the usual correlate of specific clinical deficits, since none of the cases presented cerebellar signs on imaging. This indicates functional compensation through alternative motor pathways, such as the rubrospinal tract.

## Discussion

Our case report illustrates an essential yet uncommon phenomenon of WD in bilateral MCPs following unilateral chronic PPI in an elderly woman. Ischemic strokes in the pons, often triggered by atherosclerotic changes in the large arteries supplying this region, can set off a downstream process of axonal degeneration known as WD [3]. This involves structural breakdown and disconnection of the distal ends of damaged axons, leading to scarring and atrophy of associated neural tracts [5].

WD frequently manifests after an infarct at its base in the intricate fiber network within the pons. This can symmetrically affect MCPs, which contain crossed pontocerebellar fibers originating from the contralateral pontine nuclei [19]. On MRI, WD classically appears as T2 hyperintensity and may show restricted diffusion, corresponding clinically to symptoms such as ataxia, dysmetria, and dysarthria [5,20]. While a unilateral pontine lesion can cause ipsilateral MCP WD, bilateral pontine damage often causes degeneration on both sides [19].

The precise pathological mechanisms underlying this phenomenon have been debated. One theory is that trans-synaptic degeneration occurs through the dentatorubral and dentatothalamic pathways [17,18]. Others have proposed that it may result from vasogenic edema and compression of the MCPs after extensive PPI [1,10]. However, small localized lesions have also been associated with WD, arguing against this hypothesis [1]. Recent evidence indicates a possible role for pro-inflammatory processes and macrophage activation in degenerative changes [22,23]. Axonal demyelination and disruption of neurofilaments and microtubules ultimately lead to disconnection and Wallerian-like degradation [23,24].

WD at this location has not been definitively linked to specific neurological deficits [21]. This implies compensation through alternate motor pathways, such as the rubrospinal tract. However, further studies are warranted to investigate these subtle clinical correlations. Enhancing the recognition of Wallerian-like signal changes in MCPs is vital for distinguishing these expected postinfarct changes from new ischemic lesions requiring intervention [9]. Our report provides additional documentation of this phenomenon to improve diagnostic accuracy.

Pontine lesions have diverse etiologies beyond atherosclerotic stroke, including demyelinating, inflammatory, and infectious disorders, prompting complex differential diagnoses [6]. Bilateral symmetric involvement of MCPs can also rarely occur in conditions such as adrenoleukodystrophy, PRES, and methyl bromide intoxication [8]. Therefore, the clinical context, including the risk factors and presenting symptoms, is critical. Pathological and imaging features help to refine the diagnosis. WD should be on the differential for bilateral MCP signal changes, particularly in patients with a history of PPI.

This case report had several strengths and limitations. A key strength of this study is the detailed documentation of clinical presentation, diagnostic workup with multiple imaging modalities, and clinical course over time in an elderly female patient with WD of MCPs following PPI. This report provides an excellent example of the typical features of this phenomenon on CT, MRI, and other tests. Additionally, the literature review synthesizes prior case reports on this topic to provide context and illustrate variable manifestations, imaging characteristics, and clinical implications. However, the study was limited by its focus on a single patient from a single institution. As this is an uncommon condition, more extensive scale studies across multiple sites would be beneficial to expand the knowledge of the epidemiology, risk factors, diagnostic criteria, treatment options, and outcomes. The report would also be enhanced by the patient's long-term follow-up to assess the progression of deficits and their correlation with imaging changes over time. Further neuropsychological and neurological testing could reveal subtle deficits not identified in routine examinations. Overall, this case report contributes to the literature on an important but infrequently encountered neurological complication of PPI. However, further research is needed to confirm and extend these findings.

## Conclusion

This case report describes WD of the bilateral MCP in an elderly female following unilateral chronic PPI. An ischemic stroke affecting the pons can trigger downstream axonal degeneration known as WD due to the disruption of pontocerebellar fiber crossing in this region. This is an important phenomenon to recognize in neuroimaging as it can be mistaken for new ischemic lesions. However, WD at this location has not been strongly linked to specific neurological deficits. This patient exhibited classic features of WD on MRI following a known PPI. Detailed documentation of such cases is imperative to improve diagnostic accuracy and prevent misinterpretation of these expected poststroke changes. Although an uncommon condition, knowledge of WD involving MCPs advances the clinical understanding of the secondary effects of PS. This can guide appropriate management when symmetric signal changes are observed in the neuroimaging area.

## Patient consent

A written informed consent was obtained from the patient for publication of this case and any accompanying images.
